# Effects of nicotine exposure on murine mandibular development

**DOI:** 10.1371/journal.pone.0218376

**Published:** 2019-06-13

**Authors:** E. L. Durham, C. Balog, R. N. Howie, M. A. Boyce, J. R. Arand, G. Warren, A. C. LaRue, J. J. Cray

**Affiliations:** 1 Department of Oral Health Sciences, Medical University of South Carolina, Charleston, SC, United States of America; 2 Department of Biomedical Education & Anatomy, The Ohio State University College of Medicine, Columbus, OH, United States of America; 3 Departments of Radiation Oncology and Cell and Molecular Pharmacology and Experimental Therapeutics, Medical University of South Carolina, Charleston, SC, United States of America; 4 Department of Pathology and Laboratory Medicine, Medical University of South Carolina, Charleston, SC, United States of America; 5 Ralph H. Johnson Veterans Administration Medical Center, Charleston, SC, United States of America; Charles P. Darby Children's Research Institute, UNITED STATES

## Abstract

Nicotine is known to affect cell proliferation and differentiation, two processes vital to proper development of the mandible. The mandible, the lower jaw in mammals and fish, plays a crucial role in craniofacial development. Malformation of the jaw can precipitate a plethora of complications including disrupting development of the upper jaw, the palate, and or impeding airway function. The purpose of this study was to test the hypothesis that *in utero* nicotine exposure alters the development of the murine mandible in a dose dependent manner. To test this hypothesis, wild type C57BL6 mice were used to produce *in utero* nicotine exposed litters by adding nicotine to the drinking water of pregnant dams at concentrations of 0 μg/ml (control), 50 μg/ml (low), 100 μg/ml (medium), 200 μg/ml (high) throughout pregnancy to birth of litters mimicking clinically relevant nicotine exposures. Resultant pups revealed no significant differences in body weight however, cephalometric investigation revealed several dimensions affected by nicotine exposure including mandibular ramus height, mandibular body height, and molar length. Histological investigation of molars revealed an increase in proliferation and a decrease in apoptosis with nicotine exposure. These results demonstrate the direct effects of nicotine on the developing mandible outside the context of tobacco use, indicating that nicotine use including tobacco alternatives, cessation methods, and electronic nicotine delivering products may disrupt normal growth and development of the craniofacial complex.

## Introduction

As of 2014, more than 3% of adults in the United States (US) (4% men, 3.5% women) used e-cigarettes every day or some days [1]. This is similar to the 3.4% of the US population that uses smokeless tobacco, but still pales in comparison to the 26.1% of the US population that uses smoking tobacco. Altogether, more than 1 in 4 adults in the US are regularly exposed to nicotine [2]. Nicotine, a powerful psychoactive stimulant drug and the primary compound found in tobaccos, most nicotine replacement therapeutics (NRT), as well as electronic nicotine delivering products (ENDS) has been linked to alterations of many cellular processes including cell proliferation, age-related diseases, and birth defects [3–7]. Despite the link between adverse birth outcomes of pre- and peri-natal nicotine exposure, research suggests 11% of US women continue to smoke or use alternative nicotine products through the third trimester of pregnancy [8, 9]. Nicotine has been observed to cross the placenta during pregnancy allowing for circulation and concentration in developing fetal tissues [10].

The mandible, the lower jaw in mammals and fish, plays a crucial role in craniofacial development by housing the teeth and forming an articulation with the cranium (the temporo-mandibular joint) [11]. Malformation of the jaw can precipitate a plethora of complications including disrupting development of the upper jaw and the palate, impediment of the airway, as well as altering the functional occlusion necessary for proper mastication and abnormal aesthetic appearance of the face [12]. Use of tobacco products during pregnancy has been linked to disorders of the mandible and teeth [13]. However, little work has been done to isolate the effects of nicotine in this paradigm.

As alternative pathways of nicotine use grow, including replacement therapies and ENDS, the effects of nicotine exposure may increase. Proper growth and development of the mandible requires tight control of cell proliferation, differentiation and ossification, processes nicotine exposure is known to disrupt. Thus, it stands to reason nicotine may directly affect development of the mandible. Here we investigated the direct effects of *in utero* nicotine exposure on mandibular development, hypothesizing that alterations to mandibular form will occur in a dose dependent manner.

## Methods

### Animal model and nicotine exposure

Wild type C57BL6 murine males and females (Jackson Laboratory, Bar Harbor, ME, USA) were utilized to produce *in utero* nicotine exposed litters mimicking the effects of recurrent nicotine exposure to the fetus. Nicotine (Sigma Aldrich, St. Louis, MO, USA, N3876) was added to the drinking water of pregnant dams at concentrations of 0 μg/ml (control), 50 μg/ml (low), 100 μg/ml (medium), or 200 μg/ml (high) throughout pregnancy and ceased at birth of litters. As the range of nicotine consumption by nicotine users varies widely by individual and mode of delivery, this range of doses mimics varying degrees of nicotine intake by active smokers, the largest proportion of nicotine users in the US [14–19]. Male and female mice were paired for 7 days at which point, males were removed to other pairings, or individual cages and females continued nicotine treatment until birth of the litters at ~E20. As it is unlikely that an individual would begin smoking during pregnancy, dams were pre-treated with nicotine for three weeks prior to breeding during which time, collection of blood via retro-orbital bleeding occurred weekly in the morning. Additionally, this pretreatment allowed for confirmation of nicotine consumption via assessment of the main metabolite of nicotine, cotinine. Blood serum was isolated according to manufacturer protocol (Greiner Bio-one, Kremsunster, Austria, MiniCollect Z Serum Sep.). Blood collection, and assessment of nicotine metabolism via cotinine ELISA performed according to manufacturer protocol (Calbiotech, El Cajon, CA, USA, CO096D-100) preceded breeding [20]. Animal use protocols were approved by the Medical University of South Carolina Institutional Animal Care and Use Committee (AR#3403). All breeding procedures were carried out in an Association for Assessment and Accreditation of Laboratory Animal Care International accredited facility where all husbandry and related services are provided by the Division of Laboratory Animal Resources. Both food and water, including nicotine supplemented water were made available ad libitum, and animals were monitored daily for signs of dehydration and distress. All procedures and the reporting thereof are in compliance with the Animal Research: Reporting in Vivo Experiments (ARRIVE) guidelines (**S1 Table**) [21].

### Cephalometric analysis

**Fig 1:**
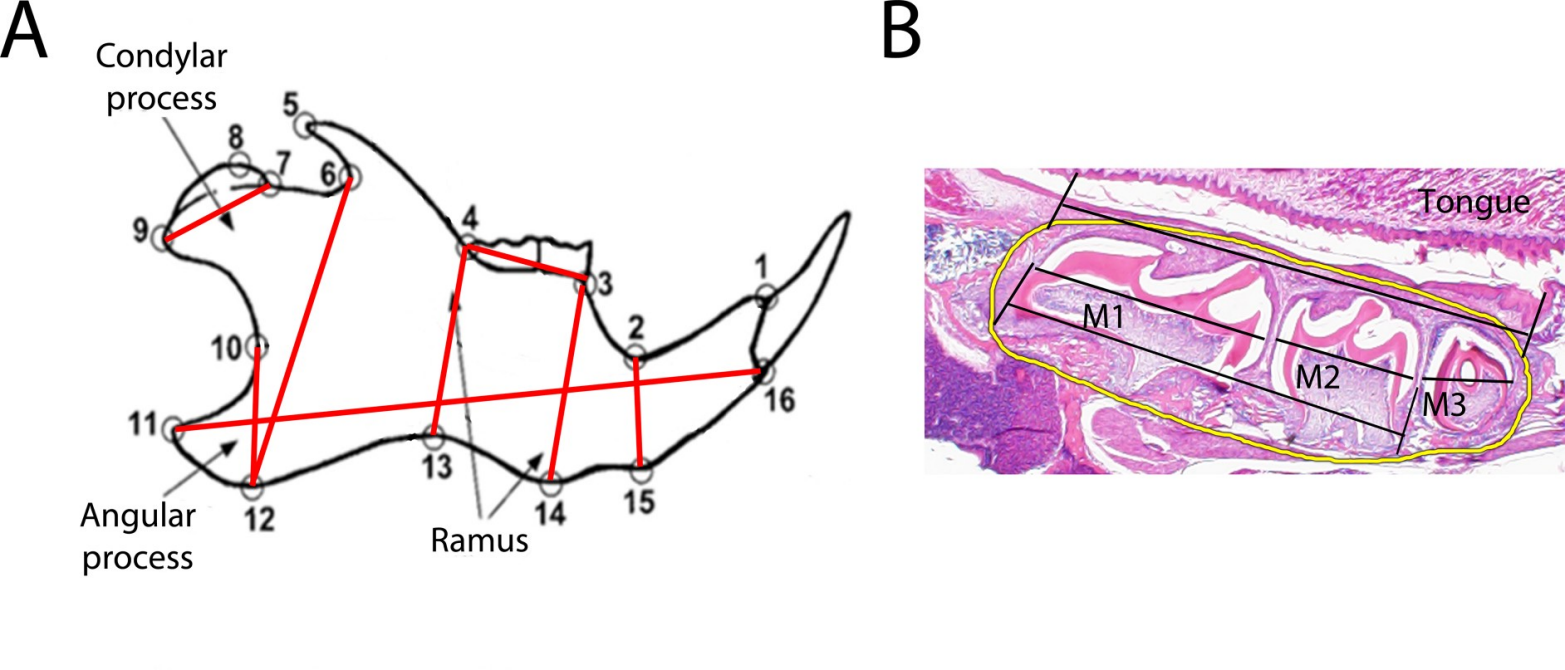
Mandible landmarks and measurement schematics. **A)** Cephalometric landmarks on murine mandible used for measurements. Dark lines between points indicate measures used in this analysis. Schematic modified from Klingenberg et al. 2004. **B)** Representative hematoxylin and eosin stained molars with lengths measured indicated with black lines, area of interest surrounding molars indicated in yellow, and molars 1–3 identified.

Pups from 2 or 3 litters resulting from the pre-treated paired breeding (**Table 1**) were grown for 15 days, the age at which reliable radiographic images can be procured for cephalometric analysis, then sacrificed using carbon dioxide asphyxiation with secondary cervical spine dislocation [17, 22, 23]. All collected skulls were fixed with 4% paraformaldehyde, then switched to 70% Ethanol and bisected along the sagittal suture. Hemi-sections were radiographed laterally using a Faxitron X-Ray imaging instrument (Faxitron X-Ray, Wheeling, IL, USA) and PPL film (Carestream, NY, USA) following initial calibration. X-Ray images were scaled using a radio opaque standard included in each image, digitized, and anatomical landmarks were identified to facilitate cephalometric analysis (**Fig 1A**) [24]. Standard linear measurements were captured using NIH ImageJ.

**Table 1:** Sex demographics and nicotine exposures.

		*In Utero* Nicotine Exposure (μg/ml)				
		0	50	100	200	Total
**Sex**	**Male**	6	7	7	7	27
	**Female**	7	5	5	6	23
**Total**		13	12	12	13	50

### Histology

Since no sex specific differences were identified in the cephalometric analysis (p = 0.737) representative (n = 4, 2 male, 2 female from separate litters) from each group (0 μg/ml (control), 50 μg/ml (low), 100 μg/ml (medium), or 200 μg/ml (high)) hemi-sected skulls were collected and decalcified in 0.25M EDTA at pH7.4 for 14 days. Samples were then wash, dehydrated in graded ethanol (70% - 100%), cleared in xylene and embedded in paraffin cut side down. Histological assessment was performed on at least three 8 μm sections at least 30 μm apart per sample for analysis of the molars. Sections were stained with hematoxylin and eosin per standard methodology.

For immunohistochemistry, slides were subjected to epitope retrieval using Tris-EDTA buffer (Caspase only) or Sodium Citrate buffer (PCNA only) and endogenous peroxidase activity block with 3% hydrogen peroxide and then washed sections were blocked in 1% goat serum with 1% bovine serum albumin. Sections were incubated with the following primary antibodies at 4°C overnight (Caspase) or 2 hours room temperature (PCNA): Proliferating Cell Nuclear Antigen (PCNA) (AbCam, Cambridge, MA, USA, ab18197, 1:3000), Active Caspase 3 (Caspase) (AbCam, ab2302, 1:75). Sections were washed and incubated with HRP conjugated secondary antibody for one hour (ab6721). Diaminobenzidine (DAB) (Vector Laboratories, Bulingame, CA) chromagen was used to identify immunoreactive structures.

All stained slides were photographed for analysis (Olympus, Miami, FL, USA, TH4-100). Measurements were conducted using CellSens imaging software (Olympus) measuring from the most anterior to most posterior aspects of each structure (**Fig 1B**). Immunohistochemical staining was quantified within the indicated area of interest surrounding the molars (**Fig 1B**) using Image J Software and the IHC Profiler Open Source Plugin for automated scoring of percent positivity [25].

### Statistics

Sex was used as a co-variate for cephalometric analyses by using a standard ANCOVA to investigate dose interactions. Bonferroni post hoc analyses were use where appropriate. For histological analyses, sex was pooled as no differences were identified between sexes in the cephalometric analysis. Standard one way ANOVA were employed to investigate the dose interactions and main effects were assessed using a standard ANCOVA to investigate dose interactions. Violations of homogeneity of variance and normality were corrected using Welch’s correction and transformations or use of non-parametric alternative tests. At least 10% of measures were repeated for each observer and all were significantly correlated. Differences were considered significant if p≤0.05. Data are presented as mean ± standard error or categorized by litter with the mean for males per exposure indicated with a black bar and the mean for females per exposure indicated by a white bar.

## Results

### *In utero* exposure

**Fig 2:**
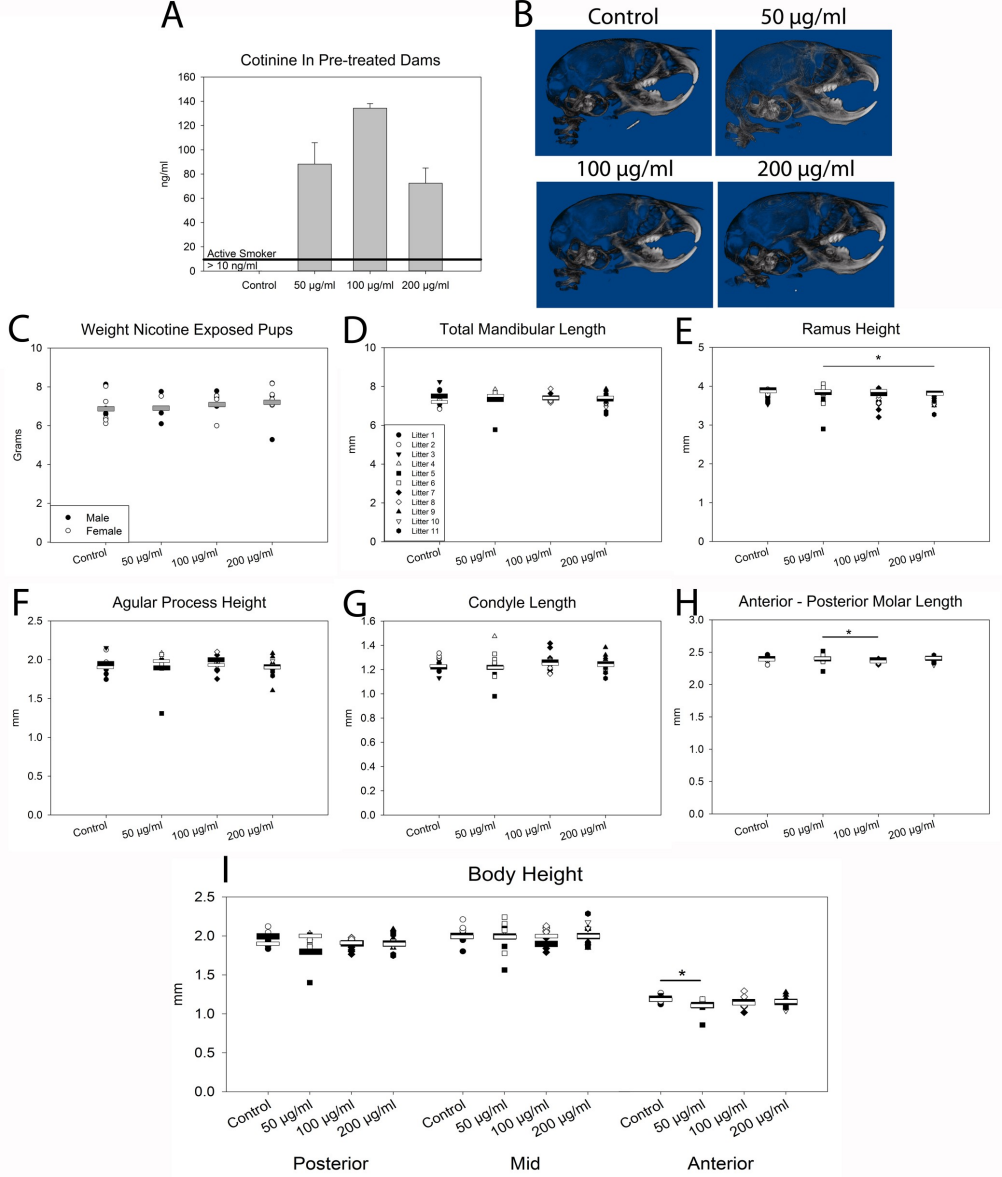
*In Utero* nicotine exposure model and cephalometric analysis of mandible. **A)** Nicotine metabolite cotinine is present at levels approximating active smoking in the blood serum of dams pretreated for 3 weeks with nicotine in drinking water. **B)** Representative lateral digital X-Rays of 0 μg/ml (control), 50 μg/ml (low), 100 μg/ml (medium), or 200 μg/ml (high) nicotine exposed post-natal day 15 mouse pups. **C)** *In utero* exposure to nicotine did not reduce weight of post-natal day 15 mouse pups as compared to control. Data categorized by sex with mean per exposure indicated with grey bar. Total mandibular length **(D)** did not vary with nicotine exposure, however, *in utero* exposure to high dose nicotine decreased the height of the ramus **(E)** significantly compared to low dose nicotine, perhaps indicating a dose dependent effect. Neither angular process height **(F)** nor condylar length **(G)** varied significantly with exposure. Molar length was significantly reduced between low and medium dose nicotine **(H)**.The height of the body of the mandible did not vary in the mid or posterior measures but was reduced with low dose nicotine (50 μg/ml compared control) anteriorly **(I)**. Data categorized by litter with mean for males per exposure indicated with black bar and mean for females per exposure indicated with white bar. *p<0.05 for differences between doses. No significant differences were identified between sexes.

Cotinine, the major metabolite of nicotine was measured in dams pretreated for three weeks with the above nicotine doses in drinking water. All nicotine exposures modeled cotinine levels mimicking active smoking (cotinine in serum >10ng/ml) (**Fig 2A**) [20]. Representative lateral X-Rays from post-natal day 15 mice exposed *in utero* to 0 μg/ml (control), 50 μg/ml (low), 100 μg/ml (medium), or 200 μg/ml (high) nicotine are included in **Fig 2B**. Craniofacial abnormalities due to exposure are subtle. Individuals per exposure are balanced between nicotine doses and sexes (**Table 1**). Weight at post-natal day 15 did not differ significantly with exposure or sex (**Fig 2C**).

### Cephalometric analysis

Landmarks and measures are identified in **Fig 1A**. When controlling for sex, total mandibular length did not vary with exposure (**Fig 2D**). Height of the ramus was decreased (p = 0.036) with the highest nicotine exposure (200 μg/ml) as compared to the lowest (50 μg/ml) (**Fig 2E**). Angular process height and condylar length did not vary with exposure (**Fig 2F and 2G**). Molar length was significantly shorter in individuals exposed to the medium (100 μg/ml) dose nicotine as compared to low (50 μg/ml) (p = 0.015) (**Fig 2H**). Neither mid nor posterior mandibular body height varied with nicotine exposure (**Fig 2I**), however anterior mandibular body height was reduced in low dose (50 μg/ml) nicotine exposed individuals as compared to control (p = 0.05) (**Fig 2I**). No significant asymmetry was observed as a result of nicotine exposure when right and left hemi-mandibles were compared for each individual by each exposure group (p = 0.174). No significant differences in any measure were identified between sexes (p = 0.737).

### Histological analysis

**Fig 3:**
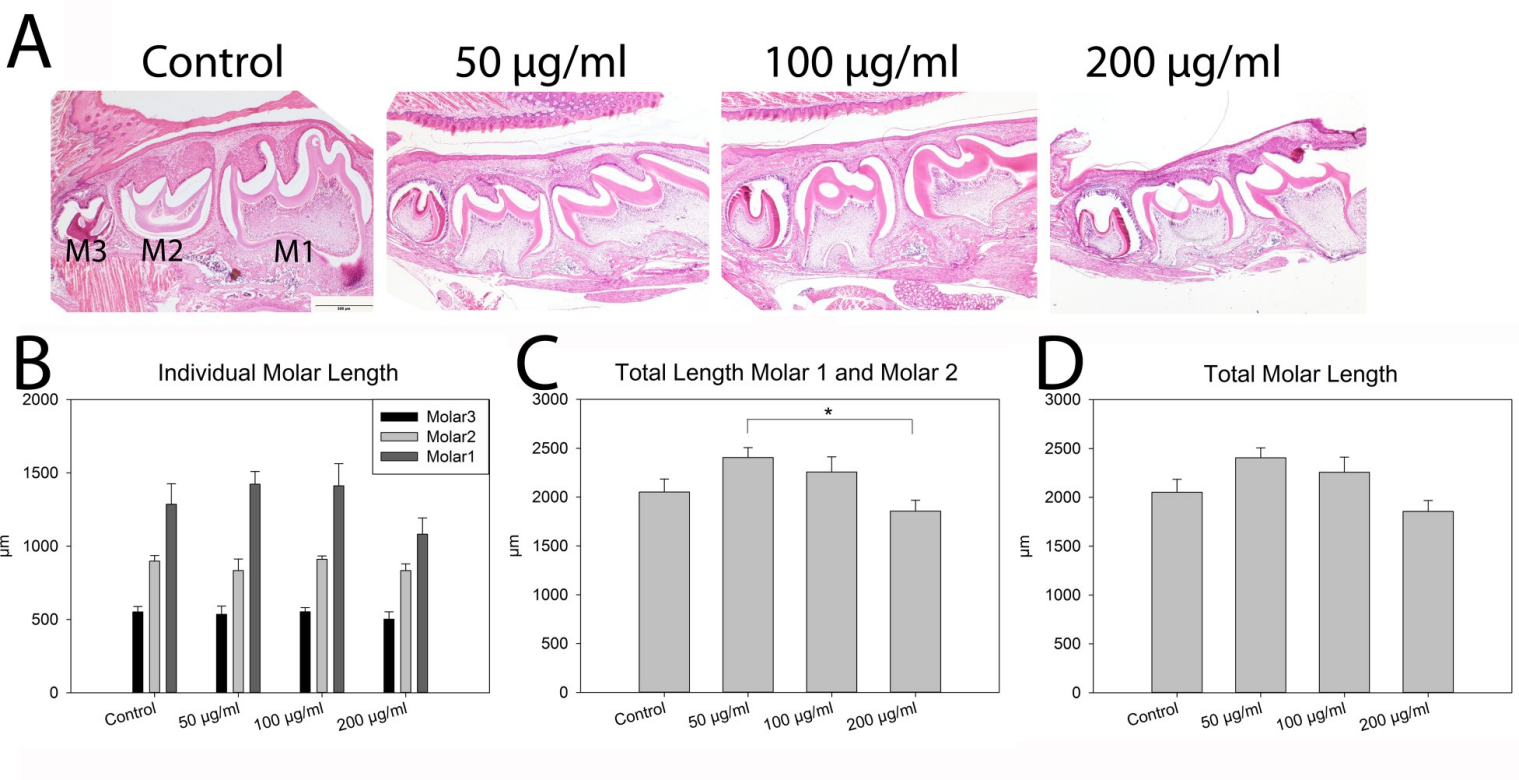
Histological analysis of mandibular molars. **A)** Representative hematoxylin and eosin stained sections of mandibular molars from individuals exposed to 0 μg/ml (control), 50 μg/ml (low), 100 μg/ml (medium), or 200 μg/ml (high) nicotine *in utero*. Molars 1–3 are marked M1, M2, M3 with tongue above for orientation. **B)** Histomorphometric analysis of molar length for M1, M2, M3 indicate no change with exposure. **C)** Length of erupted molars 1 and 2 together is reduced with the highest nicotine dose compared to the low dose however, the total molar length does not vary **(D)**. n = 4 per exposure Scale Bar = 500 μm. *p<0.05.

In order to more specifically investigate the identified abnormalities with mandibular molar development, representative specimen were sectioned, stained, imaged, and measured. Assessment of individual molars did not indicate a change in size due to nicotine exposure (**Fig 3A and 3B**). However, the combined length of molars 1 and 2 was reduced in high (200 μg/ml) dose exposed individuals as compared to low dose (50 μg/ml) (p = 0.034) (**Fig 3C**). The total length of molars 1, 2, and 3 together was not different between control and any of the nicotine doses (**Fig 3D**).

**Fig 4:**
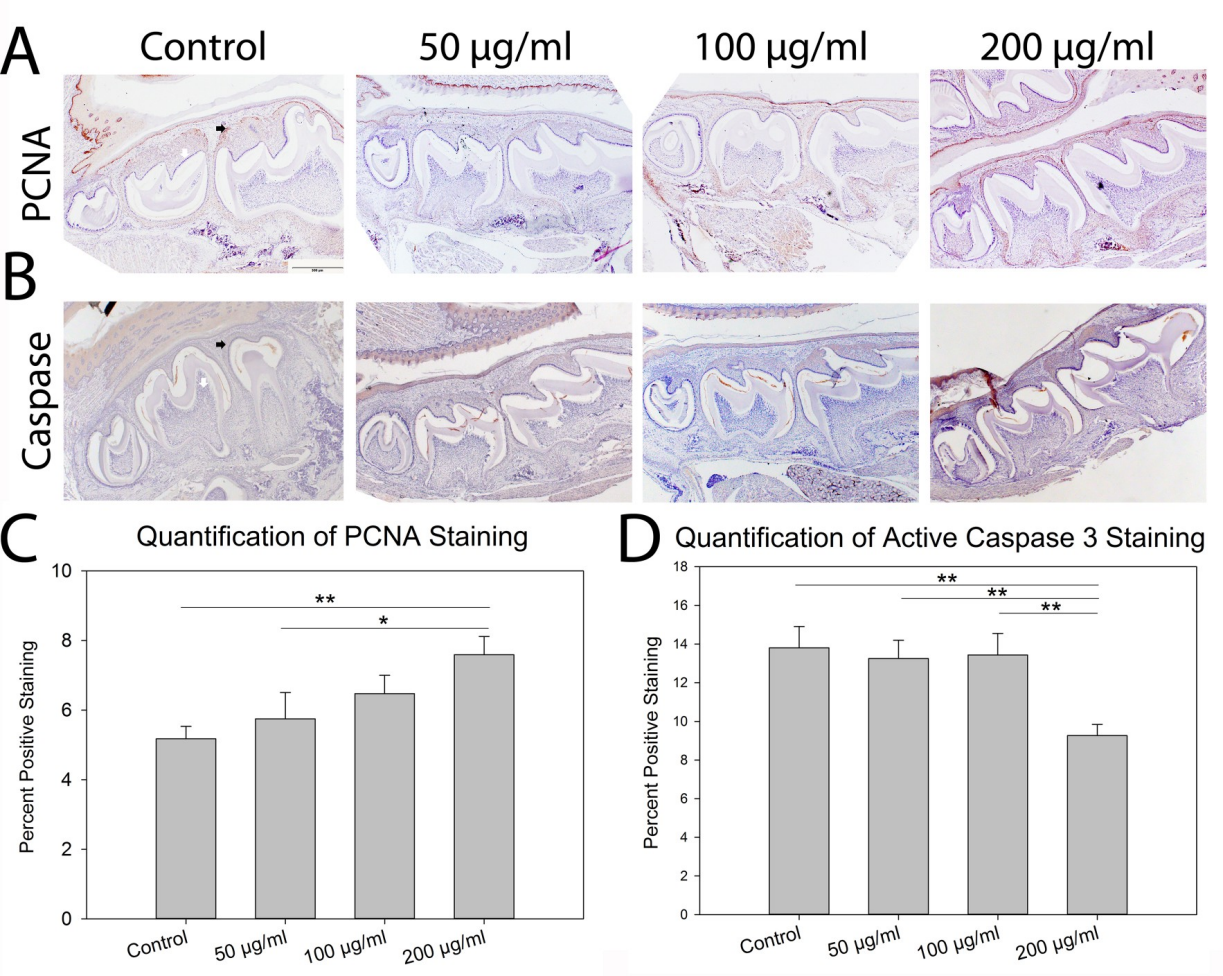
Cell proliferation and death in mandibular molars. **A-B)** Representative histological images of mandibular molars stained for Proliferating Cell Nuclear Antigen (PCNA, A) and Active Caspase 3 (Caspase, B) from individuals exposed to 0 μg/ml (control), 50 μg/ml (low), 100 μg/ml (medium), or 200 μg/ml (high) nicotine *in utero*. Black horizontal arrows indicate positive staining and white vertical arrows indicate negative cells for each target. **C)** Quantification of percent of positive staining within the area of interest surrounding the molars (**Fig 2B**) indicates increasing proliferation with nicotine exposure. **D)** Apoptosis as indicated by positive caspase staining was reduced in the area of interest surrounding the molars in individuals exposed to high dose nicotine as compared to control and all other nicotine doses. n = 4 per exposure Scale Bar = 500 μm. *p<0.05 **p<0.01.

As nicotine is known to affect cell cycle regulation [3–7], an additional assessment of cell proliferation and death in the area surrounding the molars was conducted. High dose (200 μg/ml) nicotine exposure increased proliferation (PCNA) in the area surrounding the molars as compared to control (p = 0.003) and low dose (50 μg/ml) nicotine exposure (p = 0.03) (**Fig 4A and 4C**). Apoptosis (Caspase) was reduced in individuals exposed to high dose (200 μg/ml) nicotine as compared to control (p = 0.004), medium dose (100 μg/ml) nicotine (p = 0.009) and low dose (50 μg/ml) nicotine (p = 0.002) (**Fig 4B and 4D**).

## Discussion

Our data indicate that *in utero* nicotine exposure negatively effects mandibular development, however, there was not a clear dose dependent response. Our confirmation of the exposure model by measuring the cotinine levels of the pre-treated dams highlights the vast range of cotinine levels that indicate active nicotine exposure [18, 19, 26]. The observed reduction in cotinine in the highest dose of nicotine (200 μg/ml) may indicate less consumption of water in those individuals, however animals were monitored for dehydration daily throughout the pretreatment, breeding, and pregnancy and no indication of dehydration was observed. This reduction in cotinine may also be related to variable metabolism of nicotine[17–19, 27]. Unlike in the human population, we did not observe a reduction in weight of the nicotine exposed animals 15 days post-natal, indicating that if there was a reduction in birth weight associated with *in utero* nicotine, it was regained quickly after birth in this murine model [3, 28].

The cephalometric analysis of the mandible indicated only subtle (not statistically significant) changes to the shape at this young stage of development. It has been established that effects of nicotine begin pre-natally and extend post-natally, even in the craniofacial skeleton [17, 29]. Thus, it is possible that a more dramatic effect would be observed in more aged animals due to compounding of small changes over time. Subtle changes early in development may be biologically significant though they may not be statistically significant at this timepoint. The shorter ramus, shorter anterior mandibular body, and abnormal molar length observed may be the first signs of changes in growth trajectory that can contribute to growth disturbances that compound throughout growth [30, 31]. We did not observe the predicted additive effects of increasing dosages of nicotine. The differences we have identified between nicotine doses may indicate that each dose of nicotine has specific effects, and each individual may have a different level of tolerance for this insult. Further, as we have only investigated one age of exposed individual it is possible that redundancies in the pattern of mandibular growth and development allow for compensation for this teratogenic insult.

The histological assessment of murine molars corroborated to some degree; the changes observed in the cephalometric analysis indicating that erupted molar length is reduced with nicotine exposure. This is also in agreement with research in the human population that indicates that maternal nicotine use leads to a marginal reduction in tooth size [13]. In general, we observed that the molars of individuals exposed to the highest dose of nicotine (200 μg/ml) had reduced length. It is possible that the reduction in length of the erupted molars (M1, M2) in particular is the result of a delay in molar eruption. Further, nicotine may also delay the normal cytodifferentiation required to form dentin and enamel and the rest of the complex structure of molars [29].

Differences between the cephalometric and histological assessments may be due to artifacts of processing including the necessary decalcification used to prepare samples for histological assessment. Thus, the more directed assessment of cell proliferation and apoptosis in the area of the molars provides insight into the effects of *in utero* nicotine exposure on tooth development. The significant increase in proliferation and decrease in apoptosis noted with the high dose (200 μg/ml) nicotine exposure in our study parallels other studies which indicate that nicotine potentially effects the overall shape of teeth [32]. Further, nicotine is known to negatively affect odontogenesis by delaying dentin and enamel formation and interfering with cellular processes necessary for tooth development [33]. The increase in proliferation surrounding the molars observed with nicotine exposure may be evidence of delayed tooth formation as proliferating cells may not be also differentiating appropriately. This delay may precipitate additional clinical dental issues including an increased need for orthodontics and an increased risk for caries [13, 34]. Proper tooth development relies on coordinated cell proliferations, differentiation, and apoptosis. Our data indicate that *in utero* exposure to nicotine may negatively affect this coordination leading to aberrant and potentially persistent mandibular development.

## Conclusions

These results demonstrate the direct effects of nicotine on the developing mandible, outside the context of tobacco use. The significant alteration to the growth of the ramus, body, and mandibular molars could have additional consequences as individuals continue to grow post-natally. Importantly, these data indicate that tobacco alternatives, including cessation methods and ENDS that incorporate nicotine, which may be marketed as safer than cigarette use, also disrupt normal growth and development of the craniofacial complex.

## Supporting information

S1 Table (PDF)
